# A Systematic Review of Pediatric and Adult In-Flight Medical Emergencies

**DOI:** 10.1155/2018/6596490

**Published:** 2018-11-25

**Authors:** Christina Sul, Sherif M. Badawy

**Affiliations:** ^1^Department of Pediatrics, Northwestern University Feinberg School of Medicine, Chicago, Illinois, USA; ^2^Division of Hematology, Oncology and Stem Cell Transplant, Ann & Robert H. Lurie Children's Hospital, Chicago, Illinois, USA

## Abstract

In-flight medical emergencies (IMEs) are acute onboard events of illnesses or injuries with potential immediate risk to a passenger's short- or long-term health, or life. IMEs are significant events that are related to public safety concerns. With the increasing amount of annual air travel every year, it is expected that the number of encountered IMEs will continue to grow. Thus, it will be critical to develop and implement appropriate measures to manage IMEs with the best possible outcome. Despite the fact that most IMEs are self-limited with no serious adverse events, serious IME can lead to death, disability, or other unfavorable health outcomes, particularly as a result of suboptimal medical care. In this article, we systematically reviewed the published up-to-date evidence on the subject of in-flight emergencies with a specific focus on pediatric population.

## 1. Introduction

Almost 4 billion passengers fly worldwide each year on commercial airlines [1]. While the medical field looks to aviation for key concepts of quality control and safety, ironically medical emergencies that occur on board of commercial aircraft are often chaotic events compounded by noise and vibration, limited space, lack of privacy, and language barriers. It is estimated that approximately 44,000 in-flight medical emergencies occur worldwide annually [2, 3], a number likely to increase as more passengers travel by air each year. In-flight medical emergencies (IME) are rare events; however when they do occur, access to medical care is limited by the distance to a nearest medical center, contents of an emergency medical kit, training of the onboard personnel, and availability of medically trained volunteers among other factors. Further, lack of standardized reporting of IMEs makes it difficult to perform epidemiologic research to identify areas for improvement. With increasing number of travelers overall, pediatric passenger population is also increasing; however very little is known about pediatric IMEs. Despite comprising almost 10% of the IMEs, there are only two studies to date that have been dedicated solely to pediatric IMEs [4, 5]. Our objective was to systematically review the published most recent evidence on the subject of in-flight emergencies with a specific focus on pediatric population.

## 2. Methods

### 2.1. Literature Search

This systematic review covered literature published until 2018 with no language limits. The search strategy looked for all articles on in-flight medical emergencies. We searched MEDLINE (through PubMed) from inception to May 15, 2018. We followed the Preferred Reporting Items for Systematic Reviews and Meta-Analyses (PRISMA) guidelines in the reporting of evidence across the studies reviewed herein [6]. Two independent reviewers (CS and SMB) assessed abstracts and articles against the eligibility criteria. Disagreements were resolved by discussion.

### 2.2. Eligibility Criteria

Eligible studies were original research articles, medical emergencies occurring while in-flight and on board of commercial aircraft, and articles that included pediatric patients from the analysis. We excluded studies that focused on medical education, telemedicine techniques for air-to-ground data transmission, role of medical volunteers in flight diversion decision-making, and in-airport medical emergencies.

### 2.3. Data Synthesis

We used a standardized form for data extraction. Data items in the extraction form included the following: first author's name; publication year; country; aim of the study; data source; participants' age and sex; study design and setting; sample size; selection criteria; and follow-up.

## 3. Results

### 3.1. Literature Search

**Figure 1:**
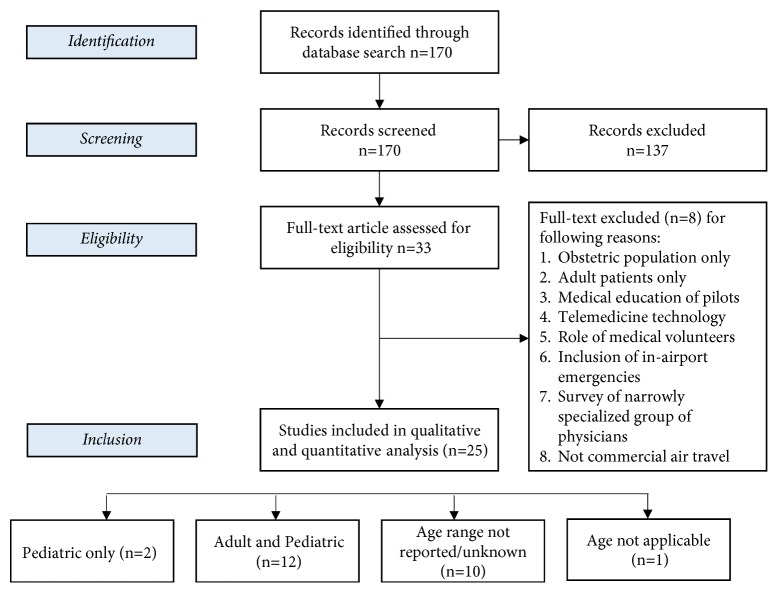
Flow of studies through the review according to the PRISMA guidelines.

A total of 170 articles were identified and retrieved. Two authors (CS and SB) independently screened the article titles and abstracts. A total of 33 records met the predefined inclusion criteria. Two authors (CS and SB) then independently reviewed the full text of these articles in detail against the exclusion criteria, and 8 items were excluded. A total of 25 articles met the predefined criteria to be included with only two articles that focused solely on pediatric in-flight emergencies. We did not identify any non-English articles that met our inclusion criteria. The selection process and reason of excluding full-text publications are outlined in an adapted PRISMA study flowchart (Figure 1) [6].

### 3.2. Study Characteristics

While our search identified articles published prior to 1980s, only articles published in 1989 and after met the predefined inclusion criteria; 4 out of 28 included studies that were published in 1989, 1 in 1990s, 14 in 2000s, and 9 in the current decade. Over half of the included studies have been performed in the United States (in whole or in part), 13/25 [2–5, 7–15], 4 in Germany [16–19], 3 in UK [20–22], 1 form Australia [23], 1 in Canada [24], 1 from China [25], 1 from France [26], and 1 in Turkey [27]. Most studies focused on characterizing IMEs on commercial aircraft. Collected and reported data commonly included patients' demographic information, type of medical or surgical complaint, utilization of the medical kits, presence of medical volunteers, and rates of diversion of the aircraft. Few studies have focused on a specific subset of patient population such as pediatric [4, 5], neurologic [15], and psychiatric [7]. 12 out of 25 included studies reporting data on both pediatric and adult patients [2, 3, 7–10, 16, 17, 20, 25–27]; ten studies either did not specify the age range of the patients' whose records were analyzed or did not include data on age altogether [11–15, 18, 21–24]; for these studies the population column in Table 1 is marked as unknown. Two articles explored pediatric-only in-flight emergencies [4, 5]. Age data was not applicable for reporting in one study; thus the population column in Table 1 is marked as N/A [19]. Overwhelming majority of the included studies are retrospective reviews that obtained data from either medical records directly reported by the commercial airlines [10, 13, 14, 17–19, 22, 23, 27], medical records kept by the airports and nearby emergency rooms and hospitals [8, 9, 14], or records collected by the air-to-ground medical support broadly categorized as telemedicine services [2–5, 7, 11, 12, 15, 20, 21, 24–26]. Two studies obtained all or part of data from questionnaires [14, 16]. Sample size ranged from 10 to 11,920, with a median of 424 and mean of 1,750 records reviewed.

### 3.3. Characteristics of Pediatric In-Flight Emergencies

Rotta et al. reported that pediatric IMEs comprise 9.3% of all in-flight emergencies or 2.24 events per 1 million passengers [4]. This number is close to 12.04% reported by Baltsezak et al. [25], 9.15% reported by Moore et al. [5], and 9% reported by Weinlich et al. [17]. Average age for pediatric in-flight emergency patient was 6.8 years reported by Moore et al. [5] and 7.3 years reported by Weinlich et al. [17]. Median age for a pediatric patient suffering a fatal event on board of commercial aircraft was 3.5 months [4]. The most common pediatric medical complains were infectious (27%), neurological (15%), and respiratory (13%) [5]. Weinlich et al. reported similar findings where infectious disease comprised 28% of pediatric in-flight emergencies, epilepsy (7%), and respiratory problems (4%) [17]. Emergency medical kit was used in 27% of pediatric in-flight emergencies, with antipyretics being the most commonly administered medication [5]. Automated external defibrillator (AED) was applied in 4 out of 10 pediatric in-flight fatalities; none of the four cases were found to have an indication for a shock [4]. In the study exploring telemedicine service for IMEs, Urwin et al. reported that the fewest air-to-ground calls were received for children 5 years and under, with only 5% calls taken for this age group [20].

### 3.4. Reporting of In-Flight Medical Emergencies

Sand et al. reported that after querying 32 commercial airlines for medical flight reports, only four (12.5%) were able to provide the required data [18]. From the included 25 studies, 6 (24%) have mentioned lack of industry-wide standardized reporting system as a factor limiting the ability to effectively access and analyze data on IMEs [3, 10, 16, 18–20].

## 4. Discussion

As the number of passengers who travel by air continues to grow, so does the incidence of IMEs. Additionally longer flight duration exposes passengers to physiologic stressors such as relative hypoxia and decreased humidity of circulating air [28]. Based on the reported data, pediatric in-flight emergencies comprise approximately 9.9% of all IMEs. Pediatric conditions that lead to diversions are neurological (due to seizures) and respiratory (due to asthma) [5], prompting a careful consideration of inclusion in emergency medical kits the weight-based seizure-aborting agents [15] and bronchodilators that can be effectively administered to young children [14]. Even when bronchodilator is available in a medical kit, the absence of appropriate delivery mechanism such as nebulizer machine or a valved holding chamber, also referred to as a spacer, makes delivery of aerosolized bronchodilator inefficient in young children [29, 30]. Presence of the nebulized bronchodilator may not be necessary on board of the aircraft as evidence has shown no significant benefit in use of the nebulizer over a metered dose inhaler (MDI) with spacer; however presence of a spacer device and appropriately sized mask is crucial to the effective delivery of the bronchodilator to the lungs [31]. Half of the passengers with respiratory symptoms suffered from an asthma exacerbation, and a third of the patients suffering an asthma attack have forgotten to bring their medication on board of the aircraft [22]. Thus, for example, a verbal or written reminder during the baggage check-in and drop-off process could be a useful tool to reduce the risk of forgetting to bring the medications on board. Further, bag-mask ventilation is a safe and effective lifesaving intervention for patients who experience critical cardiorespiratory events and require short-term ventilation support. Nevertheless, for bag-mask ventilation to be effective, the mask needs to be with the right size with the airway maintained in the optimum position and tight seal is secured around mouth and nose for effective ventilation. Although medical flight kits (MFKs) usually include bag-masks, they are usually adult-sized, making bag-mask ventilation for infants and young children not possible, if not harmful with over ventilation. So, without the right age and size-appropriate equipment onboard, available healthcare providers may not be able to provide the best case possible in these IMEs situations.

Rotta et al. focused on pediatric fatalities on board of commercial aircraft. They reported that 6 out of 10 (60%) patients suffering in-flight death did not have any preexisting medical conditions and 9 out 10 (90%) were patients under the age of 2 years, so called lap infants. Their study revealed a previously unrecognized vulnerable group of children under 2 years who share seating and/or sleeping arrangement with a potentially fatigued adult during long haul flights. Therefore, it may be helpful to have dedicated age-appropriate seats for infants under 2 years old, similar to car seats concepts, though that would come with extra cost for families.

Not enough data is available to draw objective conclusions for AED use in pediatric IMEs. However, data in adult population strongly supports presence of AED on board of commercial aircraft [13, 32]. AED use was a factor most strongly associated with diversion [2], and an installment of AEDs on a commercial aircraft has been found to be cost-effective by the conventional standards of cost-effectiveness [33].

Aside from defibrillator, the contents of the emergency medical kits are mainly determined by the consensus opinion of different airlines rather than evidence. Thus an opportunity exists to study and develop evidence-based emergency medical kits. The contents of MFKs vary significantly across different airlines, and some airlines were found to be poorly equipped to manage IMEs. While MFKs usually contain bag-masks, these are often adult-sized masks only, limiting the benefits of bag-mask ventilation for infants and children during IMEs. Given that IMEs are more likely to happen during the long haul flights, even with timely diversion, and that the arrival to a ground emergency care facility may be significantly delayed, expanding the contents of the MFKs to be suitable for Trans-Atlantic and Trans-Pacific flights is worth considering.

Further, the commercial air travel industry lacks a standardized industry-wide approach to reporting IMEs. Sand et al. argue that this lack of standardization in reporting hampers epidemiologic research [18, 19]. This notion is supported by multiple other articles included in this review [3, 10, 16, 20]. Epidemiologic research of in-flight emergencies is necessary to establish preflight screening guidelines, standardize the contents of the emergency medical kit, establish guidance for air-to-ground medical consultation, and standardize reporting of IMEs.

Our systematic review has a few strengths. First, we followed the recommendations for rigorous systematic review methodology [6, 34–37]. Second, we conducted a review with a prespecified search strategy and no language restrictions so as to minimize publication bias and identify the largest possible number of relevant studies. Third, 2 authors completed the review process independently at all stages of the systematic review. Our systematic review has two possible methodological limitations. First, similar to other systematic literature reviews, it is possible that we could have missed few relevant articles. Second, to identify the strongest most up-to-date available evidence, we included articles that were published in peer-reviewed journals, which could have introduced publication bias [38, 39].

## 5. Conclusions

IMEs are significant events that are related to public safety concerns. There is wide variability in the available services, expertise, and supplies required for the optimal management of infants and children with different IMEs. Additional help or access to resources needed onboard is often limited, which makes the situation of pediatric IMEs more complex. Having an enhanced MFK onboard could benefit pediatric patients with IMEs, particularly infants and young children, which could lead to less frequent flight diversions of flights and might have some cost benefit to airlines as well. Most flight crewmembers and staff are trained in first aid and basic life support, but lack skills needed for pediatric emergencies. This lack of preparedness further highlights the challenge of managing pediatric IME. Given the lack of standard reporting of IMEs, the problem of IMEs could be more significant since the currently used methods may have underestimated the true incidence and burden of IMEs. Central registries and documentation protocols are warranted to facilitate aeromedical research of IMEs. Well-equipped MFKs should be available in all commercial airlines onboard to optimize the management of IMEs, particularly pediatric ones. Standard regulations on the content of MFKs, supported by national and international authorities and organizations, are essential to ensure passenger safety, in particular for infants and young children.

## Figures and Tables

**Table 1:** Summary of included studies that focused on in-flight medical emergencies.

Source (country)	Purpose of the study	Study design	Population	Age, sex	Group (n)	Source of data	Duration
Moore et al. (USA)	To determine the incidence and character of pediatric emergencies on commercial airline and to evaluate current in-flight medical kits.	Observational retrospective review	Pediatric	Mean patient age 6.8 years, Sex unknown	222	Telemedicine database	7 years

Rotta et al. (USA)	To characterize in-flight pediatric fatalities onboard commercial airlines	Retrospective cohort study	Pediatric	Median patient age 3.5 months (range 1mo-15 yrs) Female 50%	10	Telemedicine database	3.5 years

Hinkelbein et al. (Germany)	To gather data on incidence, nature, and medical equipment use for in-flight emergencies	Retrospective analysis of secondary data (survey)	Adult and Pediatric	Mean patient age 54.1 years (range 15-79 yrs) Female 40.7%	54	Physician members of an aerospace medical society	1 month

Szmajer et al. (France)	To analyze the causes and outcomes of in-flight emergency calls from a commercial airline	Retrospective study	Adult and Pediatric	Mean patient age 41.12 years (range 1- 84 yrs) Female 37.7%	380	Telemedicine database	1 year

Matsumoto et al. (USA)	To ascertain the incidence of in-flight psychiatric emergencies, their associated factors, and outcomes.	Retrospective study	Adult and Pediatric	Mean patient age 39.6 years (range 10- 80 yrs) Female 73%	1,375	Telemedicine database	1 year

Kesapli et al. (Turkey)	To evaluate the incidence and status of urgent medical conditions, the attitudes of health professionals, the adequacy of medical kits and training of cabin crew	Retrospective cohort study	Adult and Pediatric	Median patient age 45 years (range 0- >=86 yrs) Female 53.2%	1,312	Commercial airline database	3 years

Peterson et al. (USA)	To describe in-flight medical emergencies and the outcomes of these events.	Retrospective cohort study	Adult and Pediatric	Mean patient age 48 years (range 14 days-100 yrs) Sex unknown	11,920	Telemedicine database	2.8 years

Weinlich et al. (Germany)	To identify which patients require diversion due to a medical incident on a commercial flight	Prospective study	Adult and Pediatric	Mean patient age 58 years (range <1 – 91 yrs) Sex unknown	3,364	Commercial airline database	3 years

Urwin et al. (UK)	To characterize medical emergencies on board of commercial flights	Retrospective review	Adult and Pediatric	Mean patient age 42 years (range 1- 87 yrs) Female 56%	273	Telemedicine database	5 years

Chan et al. (USA)	To determine incidence, post-flight treatments, outcomes, morbidity, and mortality of in-flight medical emergencies	Retrospective study	Adult and Pediatric	Mean patient age 46.6 years Female 54.7%	744	EMS, ED, and inpatient hospital records of in-flight medical emergencies from a major international airport	1 year

Speizer et al. (USA)	To evaluate the potential usefulness of any emergency medical kit	Retrospective study	Adult and Pediatric	Age unknown Female 50.8%	260	ER, hospital, airport first-aid station medical records	6 months

Delaune et al. (USA)	To determine the incidence of each type of in-flight medical complaint, the appropriateness of medical kit contents, which factors lead to aircraft diversion, and which factors affect the appropriateness of the decision to divert	Observational retrospective review	Adult and Pediatric	Not reported	2,279	Telemedicine database	1 year

Baltsezak (China)	To characterize medical emergencies on board of commercial flights	Retrospective study	Adult and Pediatric	Not reported	191	Telemedicine database	1 year

Hordinsky (USA)	To characterize in-flight medical emergencies and efficacy of medical kit usage	Retrospective review	Adult and Pediatric	Not reported	1,016	Commercial airline database	1 year

Alves et al. (USA)	To gather data on outcomes and prognostic factors for victims of in-flight cardiac arrest	Retrospective cohort study	Adult	Mean patient age 61.9 years (SD 15.62) Female 36.5%	394	Telemedicine database	10 years

Brown et al. (USA)	To describe the characteristics and outcomes of AED use during in-flight emergencies	Retrospective descriptive study	Adult	Mean patient age 58 years Female 36%	169	Telemedicine database	4.7 years

Hung et al. (UK)	To provide description of in-flight medical emergencies and systematically examine predictors of medical flight diversions and deaths	Retrospective cohort study	Adult	Age unknown, Female 53.9%	4,068	Telemedicine database	5 years

Page et al. (USA)	To analyze the AED use on board of a commercial flight	Retrospective cohort study	Adult	Mean patient age 58 years Female 34%	200	Commercial airline database	2 years

Cottrell et al. (USA)	To analyze the effectiveness of enhanced medical kit in treating in-flight emergencies on board of a commercial flight	Retrospective review and survey study	Adult	Mean patient age 54 years (males) 48 years (females) Female 43%	362	Commercial airline database, hospital records, and patient and health care providers questionnaires	1 year

Valani et al. (Canada)	To determine the causes of in-flight medical diversions from a commercial airline	Retrospective review	Not reported	Not reported	220	Telemedicine and commercial airline databases	5 years

Quereshi et al. (UK)	To analyze medical emergencies on board of single commercial airline	Retrospective review	Not reported	Not reported	507	Commercial airline database	6 months

Sirven et al. (USA)	To analyze the frequency of neurologic events during commercial airline flights and to assess whether onboard emergency medical kits are adequate for such events	Retrospective study	Not reported	Not reported	2,042	Telemedicine database	5 years

Donaldson et al. (Australia)	To analyze the types of medical emergencies encountered during commercial air travel	Retrospective study	Not reported	Not reported	454	Commercial airline database	1 year

Sand et al. (Germany)	To document medically relevant emergencies in airline passengers	Retrospective study	Unknown	Unknown	10,189	Commercial airline database	6 years

Sand et al. (Germany)	To analyze different forms of documentation for in-flight medical emergencies use by commercial airlines	Documentation analysis	N/A	N/A	10 airlines	In-flight medical emergency documentation forms from commercial airlines	Unknown
